# Association of White Blood Cell Subtypes and Derived Ratios with a Mortality Outcome in Adult Patients with Polytrauma

**DOI:** 10.3390/healthcare10081384

**Published:** 2022-07-25

**Authors:** Cheng-Shyuan Rau, Shao-Chun Wu, Ching-Hua Tsai, Sheng-En Chou, Wei-Ti Su, Shiun-Yuan Hsu, Ching-Hua Hsieh

**Affiliations:** 1 Department of Plastic Surgery, Kaohsiung Chang Gung Memorial Hospital, Chang Gung University College of Medicine, Kaohsiung 83301, Taiwan; ersh2127@adm.cgmh.org.tw; 2 Department of Anesthesiology, Kaohsiung Chang Gung Memorial Hospital, Chang Gung University College of Medicine, Kaohsiung 83301, Taiwan; shaochunwu@gmail.com; 3 Department of Trauma Surgery, Kaohsiung Chang Gung Memorial Hospital, Chang Gung University College of Medicine, Kaohsiung 83301, Taiwan; tsai1737@cloud.cgmh.org.tw (C.-H.T.); athenechou@gmail.com (S.-E.C.); s101132@adm.cgmh.org.tw (W.-T.S.); ah.lucy@hotmail.com (S.-Y.H.)

**Keywords:** trauma, injury severity score (ISS), neutrophil-to-lymphocyte ratio (NLR), monocyte-to-lymphocyte ratio (MLR), platelet-to-lymphocyte ratio (PLR), mortality

## Abstract

**Background**. After trauma, the subtypes of white blood cells (WBCs) in circulation and the derived neutrophil-to-lymphocyte ratio (NLR), monocyte-to-lymphocyte ratio (MLR), and platelet-to-lymphocyte ratio (PLR) may undergo relative changes and reflect the patients’ immune-inflammatory status and outcome. This retrospective study was designed to investigate the relationship between these variables and the mortality outcomes in adult patients with polytrauma, which is defined as an abbreviated injury scale (AIS) score ≥ 3 in two or more different body regions. **Methods**. A comparison of the expression of subtypes of WBCs, NLR, MLR, and PLR upon arrival to the emergency department was performed in selected propensity score-matched patient cohorts created from 479 adult patients with polytrauma between 1 January 2015 and 31 December 2019. A multivariate logistic regression analysis was used to identify the independent risk factors for mortality. **Results**. There were no significant differences in monocyte, neutrophil, and platelet counts, as well as in MLR, NLR, and PLR, between deceased (*n* = 118) and surviving (*n* = 361) patients. In the propensity score-matched patient cohorts, which showed no significant differences in sex, age, comorbidities, and injury severity, deceased patients had significantly higher lymphocyte counts than survivors (2214 ± 1372 vs. 1807 ± 1162 [106/L], respectively, *p* = 0.036). In addition, the multivariate logistic regression analysis revealed that the lymphocyte count (OR, 1.0; 95% confidence interval [CI], 1.00–1.06; *p* = 0.043) was a significant independent risk factor for mortality in these patients. **Conclusions**. This study revealed that there was no significant difference in the counts of monocytes, neutrophils, and platelets, as well as in MLR, NLR, and PLR, between deceased and surviving patients with polytrauma. However, a significantly higher lymphocyte count may be associated with a worse mortality.

## 1. Introduction

Polytrauma is a specific category of trauma wherein patients who have multiple injuries in the body have a life-threatening condition that is associated with potentially compromised physiology and dysfunction of uninjured organs [1]. The abbreviated injury scale (AIS), which scores and classifies injury according to six levels of severity, namely, (1) minor, (2) mild, (3) serious, (4) severe, (5) critical, and (6) mortal [2], has been used as the most relevant scale to indicate injury severity in different body regions. In addition, the injury severity score (ISS) describes injury severity through the sum of the squares of AIS of the three most severe injuries [3]. Although some data exist, the polytrauma proposed by Butcher et al., defined as an injury with AIS ≥ 3 points in at least two different body regions, has been widely used [4]. This definition includes the greatest percentage of the worst outcomes and a significantly larger percent of the clinically diagnosed polytrauma patients than those diagnosed according to the definition solely using the ISS. With a higher riskof mortality, more frequent intensive care unit (ICU) admissions, and longer stays in the hospital and ICU, AIS ≥ 3 for at least two body regions of a patient represents a better definition for polytrauma when compared with other definitions, i.e., ISS > 15, >16, or >17 [4,5,6].

When treating critically injured patients, it is important to identify those who are at high risk of mortality. The major determinants of outcome are expected to be the intensity of trauma injury to the subject and the immunoinflammatory response of the host [7,8]. Systemic immunoinflammatory response following severe injury may lead to immunological disturbances and increase the risk of sepsis and mortality. For those with polytrauma, such a systemic immunoinflammatory response may be exaggerated under such a high magnitude of injury. In trauma patients, some readily available parameters originating from a routine complete blood count, including red blood cells, white blood cells (WBCs), platelets, and information on subtypes of WBCs, as well as the derived neutrophil-to-lymphocyte ratio (NLR), monocyte-to-lymphocyte ratio (MLR), and platelet-to-lymphocyte ratio (PLR), have been investigated as potential biomarkers, with mixed results. The subtypes of WBCs may undergo relative changes under stress or systemic inflammation; thus, the derived ratios may also reflect the patients’ immune-inflammatory status and indicate their outcomes [9,10]. NLR, MLR, and PLR have been widely investigated as new inflammatory markers in many inflammatory, cardiovascular, and malignant diseases [11,12,13,14,15,16,17,18,19,20], and have been thought to be simple, inexpensive, and easily assessed predictors.

Under the hypothesis that the patients’ subtypes of WBCs and the levels of derived ratios of NLR, MLR, and PLR may reflect the host immune-inflammatory response following major trauma, and therefore be associated with the mortality outcomes of the patients, this study aimed to investigate whether these variables found in adult polytrauma patients upon arrival at the emergency room are associated with the mortality outcomes of these patients.

## 2. Materials and Methods

### 2.1. Enrolled Study Population

**Figure 1:**
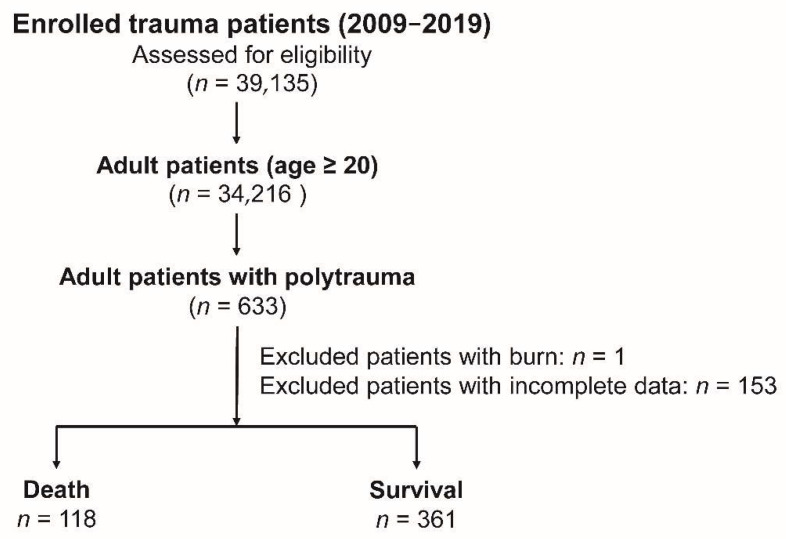
Flowchart of the process for enrolling adult patients with polytrauma.

This study was approved by the Institutional Review Board (IRB) of Chang Gung Memorial Hospital (approval numbers: 202100301B0 and 202100761B0). The need for informed consent was waived by IRB regulations owing to the retrospective nature of the registered data. Of the 34,216 adult trauma patients aged ≥20 years selected from 39,135 hospitalized trauma patients registered in the Trauma Registry System of the hospital between 1 January 2015 and 31 December 2019 [21,22,23], 633 with AIS ≥ 3 in two or more different body regions were considered to have polytrauma (Figure 1). The exclusion criteria included those patients with burns (*n* = 1) and those with incomplete data on the WBC subpopulations and platelets (*n* = 153). Finally, 479 patients were included in the study.

### 2.2. Collection of Medical Data

Medical data were extracted from the registered data from the trauma registry system, including information on sex; age; count of monocyte, neutrophil, lymphocyte, and platelets; pre-existing comorbidities (cerebrovascular accident, hypertension (HTN), coronary artery disease, congestive heart failure, diabetes mellitus, and end-stage renal disease); Glasgow Coma Scale (GCS); and ISS. The laboratory data of the subtypes of WBCs and platelets in patients upon arrival at the emergency room were collected, and the derived MLR, NLR, and PLR were calculated by dividing the monocyte count, neutrophil count, and platelet count by the lymphocyte count, seperately, with those cell counts expressing a concentration of 10^6^/L. The primary outcome of this study was the in-hospital mortality rate.

### 2.3. Statistical Analyses

All statistical analyses were performed using IBM SPSS Statistics for Windows (version 23.0; IBM Corp., Armonk, NY, USA). Two-sided Fisher exact or Pearson chi-square tests were used to compare the categorical data by calculating the odds ratio (OR) and 95% confidence interval (CI). Levene’s test was used to assess the homogeneity of variance in the continuous variables. Normally distributed continuous data were analyzed using unpaired Student’s *t*-test and are presented as mean ± standard deviation. Non-normally distributed data, such as GCS and ISS, were analyzed using the Mann–Whitney U-test and are presented as the median and interquartile range (IQR, Q1–Q3). To minimize the confounding effects of the baseline covariates that may be related to the assessment of MLR, NLR, PLR, and WBC subpopulations, propensity scores were estimated using multiple logistic regression analysis with adjustments for patient sex, age, pre-existing comorbidities, and ISS, and a 1:1 matched study group was created using the greedy method and a 0.2 caliper width using NCSS software (NCSS 10; NCSS Statistical software, Kaysville, UT, USA). Univariate predictive variables that resulted in patient mortality were identified, and multivariate logistic regression analysis was used to identify independent risk factors for mortality. Statistical significance was set at *p* < 0.05.

## 3. Results

### 3.1. Patient and Injury Characteristics of Deceased and Surviving Patients with Polytrauma

Of the 479 patients with polytrauma, 118 died and 361 survived (Table 1). There was no significant difference in sex, age, or prevalence for pre-existing comorbidities, except for a significantly lower rate of hypertension in patients who died compared with those who survived (19.5% vs. 30.7%, *p* = 0.018). Compared with those survival patients, the deceased patients had a significant higher rate of sustaining injuries to the head/neck (83.1% vs. 70.4%, *p* = 0.007) and thorax region (78.0% vs. 66.5%, *p* = 0.019), but a lower rate of injury to the extremities (38.1% vs. 50.7%, *p* = 0.018). A significantly lower GCS score (median [IQR, Q1–Q3]: 3 [3–8] vs. 13 [7–15], respectively; *p* < 0.001) but higher ISS (36 [28–41] vs. 27 [22–34], respectively; *p* < 0.001) were found in patients who died compared with in those who survived. Stratification of ISS revealed that, among the deceased patients, fewer patients had an ISS of 16–24 and more patients had an ISS of ≥25 compared with the surviving patients. There was no significant difference in the counts of monocytes, neutrophils, platelets, and lymphocytes, as well as in MLR, NLR, and PLR, between the deceased and surviving patient groups.

### 3.2. Comparison in the Propensity Score-Matched Patient Cohorts

A comparison was performed in 88 well-balanced pairs of propensity-score-matched patient cohorts (Table 2). In these propensity score-matched patients, there were no significant differences in sex, age, comorbidities, and ISS. The comparison in these propensity score-matched patient cohorts revealed that there was no significant difference in the counts of monocytes, neutrophils, and platelets, as well as in MLR, NLR, and PLR, between the deceased and surviving patients. However, deceased patients had a significantly higher lymphocyte count than survivors (2214 ± 1372 vs.1807 ± 1162 [10^6^/L], respectively, *p* = 0.036).

### 3.3. Analysis of Risk Factors for Mortality

The univariate analysis revealed that the HTN, GCS score, and ISS were significant risk factors for mortality in adult patients with polytrauma (Table 3). The subsequent multivariate logistic regression analysis revealed that age (OR, 1.0; 95% CI, 1.01–1.05; *p* < 0.001), HTN (OR, 0.5; 95% CI, 0.26–0.98; *p* = 0.042), GCS (OR, 0.8; 95% CI, 0.76–0.86; *p* < 0.001), ISS (OR, 1.1; 95% CI, 1.07–1.14; *p* < 0.001), and lymphocyte count (OR, 1.0; 95% CI, 1.00–1.06; *p* = 0.043) were significant independent risk factors for mortality in these patients.

## 4. Discussion

Among the subtypes of WBCs, neutrophils are the most abundant and act as the key cell type of the innate immune system, as the first cellular line of the defense system. Monocytes are essential components of the innate immune system and act with antigen presentation to affect the adaptive immune system. In addition, lymphocytes are involved in adaptive immune responses. Trauma is expected to affect the immune defense mechanism, and it may not be surprising to find that the circulating subtypes of WBCs would not only respond to pathogenic organisms in sepsis patients, but also to the stress associated with severe trauma. Severe trauma has been reported to result in the systemic release of pro-inflammatory cytokines and inflammatory mediators into circulation with profound acute-phase responses [24,25]. The activation of neutrophils with an intensified acute inflammatory response induces hyperinflammatory responses and tissue damage, with an increased production of tumor necrosis factor-α, interleukin (IL)-1, and IL-6, as well as an uncontrolled burst of polymorphonuclear cells and macrophages [26,27,28]. Monocytes can differentiate into activated macrophages, regulate inflammatory responses through IL-10 and transforming growth factor-β1 production, and promote tissue repair [29]. Under severe trauma, the deactivation of monocytes is correlated with the injury severity of the patients [30], leading to immunocompetence of the cells with a profound loss of antigen-presenting ability [31]. However, although MLR, NLR, and PLR have been implicated as useful biomarkers for trauma patients in some studies [32,33,34,35], this study of propensity score-matched patient populations with polytrauma revealed that there was no significant difference in the counts of monocytes, neutrophils, and platelets, as well as in MLR, NLR, and PLR between patients who died and survived. In contrast, patients who died had a significantly higher lymphocyte count than the survivors. Furthermore, the multivariate analysis also revealed that the lymphocyte count was a significant independent risk factor for mortality in patients with polytrauma. In our prior study of trauma patients in the ICU, we demonstrated that a higher lymphocyte count, lower platelet count, and lower PLR were associated with a higher risk of death in ICU trauma patients [32]. We believe that, unlike some studies [28,29,30,31], MLR, NLR, and PLR failed to be associated with the mortality outcome, probably because they were assessed in more severely injured subjects, i.e., polytrauma patients. In the case of profound injury, the difference in the ratio between deceased and surviving patients could be reduced and less remarkable. The study population may determine the usefulness of the ratio of WBC subtypes applied in the clinical setting.

In this study, the deceased patients of the matched cohorts had a significantly higher lymphocyte count than the surviving patients (2214 ± 1372 vs.1807 ± 1162 [10^6^/L], respectively, *p* = 0.036). Univariate and multivariate analyses showed that the lymphocyte count (OR, 1.0; 95% CI, 1.00–1.06; *p* = 0.043) was a significant independent risk factor for mortality, although the odds of difference was quite minimal. These results are in accordance with those reported by Riché et al. [36] in a prospective, single-center, observational study of 130 patients, in which the neutrophil count upon admission was similar between survivors and non-survivors, while the lymphocyte count was higher in non-survivors. However, the results seem to contradict those reports that lymphopenia was observed within 24 h of injury [37], persistent in those who developed multiple organ dysfunction [37], and was associated with increased mortality [38,39]. In a 2-week longitudinal study characterizing lymphocyte response patterns following severe trauma, there was dramatic activation of the circulating lymphocytes, which are generally independent of the clinical course [40]. It was reported that, after severe trauma, ISS had a negative correlation with a population of CD3(+) T cells [41]. T lymphocytes were activated following trauma, with a subsequent significant reduction in the percentage and absolute number of lymphocytes in comparison with the control [40]. In a study of 105 trauma patients with an ISS of 20 or greater, significant lymphopenia occurred in the first post-injury week, with a maximal reduction at 3 days [39]. There is an estimated 45% mortality rate in patients with a lymphocyte count of ≤500 × 10^6^/L at 48 h [37]. Regarding the lymphocyte count and mortality outcome, we believe that, in this study, the detection of the blood profile immediately following trauma, but not days later, as in other studies, and the high injury severity of the study population may account for the difference in the observed outcome between the present study and some of the other studies. Therefore, monitoring the dynamic changes in immune cells after polytrauma may be necessary to collect more information, which is one important limitation when interpreting the results of this study.

This study also had other limitations. First, selection bias may exist owing to the retrospective study design and after excluding incomplete data. For example, the trauma database did not record patients who were declared dead on arrival at the emergency room, and only in-hospital mortality was assessed; therefore, selection bias may have existed during data analysis. Second, the platelet and WBC subtypes may interfere with resuscitation procedures, such as blood transfusion or fluid challenge, leading to a bias in the outcome measurement. Third, invasive procedures and surgery could have led to different patient outcomes; however, we can only assume that the management had uniform outcomes across the studied patients. Furthermore, this study cannot account for the characteristics and mechanisms underlying the changes in lymphocytes. Finally, the population in this study was limited to a single urban trauma center, and the results may not be generalizable to other regions.

## 5. Conclusions

This study revealed that a significantly higher lymphocyte count in adult patients with polytrauma upon arrival at the emergency room was associated with worse mortality outcomes. However, there was no significant difference in the counts of the monocytes, neutrophils, and platelets, as well as in MLR, NLR, and PLR between deceased and surviving patients.

## Figures and Tables

**Table 1:** Patient and injury characteristics of the deceased and surviving patients with polytrauma.

Variables	Death *n* = 118		Survival *n* = 361		OR (95% CI)	*p*
Male, *n* (%)	87	(73.7)	234	(64.8)	1.5 (0.96–2.42)	0.074
Age, age	55.0	±20.7	56.5	±20.2	-	0.487
Comorbidities						
CVA, *n* (%)	1	(0.8)	10	(2.8)	0.3 (0.04–2.37)	0.226
HTN, *n* (%)	23	(19.5)	111	(30.7)	0.5 (0.33–0.91)	0.018
CAD, *n* (%)	6	(5.1)	18	(5.0)	1.0 (0.40–2.64)	0.966
CHF, *n* (%)	0	(0.0)	1	(0.3)	-	0.567
DM, *n* (%)	14	(11.9)	61	(16.9)	0.7 (0.36–1.23)	0.192
ESRD, *n* (%)	0	(0.0)	6	(1.7)	-	0.159
GCS, median (IQR)	3	(3–8)	13	(7–15)	-	<0.001
Injured region, AIS ≥ 3						
Head/Neck, n(%)	98	(83.1)	254	(70.4)	2.1 (1.21–3.51)	0.007
Face, *n* (%)	5	(4.2)	9	(2.5)	1.7 (0.57–5.27)	0.329
Thorax, *n* (%)	92	(78.0)	240	(66.5)	1. 8 (1.10–2.90)	0.019
Abdomen, *n* (%)	26	(22.0)	88	(24.4)	0.9 (0.53–1.44)	0.604
Extremity, *n* (%)	45	(38.1)	183	(50.7)	0.6 (0.39–0.92)	0.018
External, *n* (%)	0	(0.0)	1	(0.3)	-	0.567
ISS, median (IQR)	36	(29–41)	27	(22–34)	-	<0.001
16–24	4	(3.4)	110	(30.5)	0.1 (0.03–0.22)	<0.001
≥25	114	(96.6)	251	(69.5)	12.5 (4.50–34.70)	<0.001
Monocytes (10^6^/L)	556	±361	576	±358	-	0.599
Neutrophils (10^6^/L)	8899	±6020	9331	±5019	-	0.441
Platelets (10^6^/L)	232,805	±75,994	221,967	±68,481	-	0.147
Lymphocytes (10^6^/L)	2130	±1311	1996	±1534	-	0.394
MLR	0.4	±0.4	0.4	±0.3	-	0.746
NLR	6.9	±10.4	6.7	±5.6	-	0.829
PLR	149.4	±100.8	146.2	±80.1	-	0.728

CAD = coronary artery disease; CHF = congestive heart failure; CI = confidence interval; CVA = cerebral vascular accident; DM = diabetes mellitus; ESRD = end-stage renal disease; GCS = Glasgow Coma Scale; HTN = hypertension; IQR = interquartile range; ISS = injury severity score; MLR = monocyte-to-lymphocyte ratio; NLR = neutrophil-to-lymphocyte ratio; OR= odds ratio; PLR = platelet-to-lymphocyte ratio.

**Table 2:** Comparison of the neutrophil-to-lymphocyte ratio, monocyte-to-lymphocyte ratio, platelet-to-lymphocyte ratio, and subtypes of white blood cells in propensity score-matched cohorts of patients with polytrauma.

Variables	Propensity Score-Matched Patient Cohorts						
	Death *n* = 88		Survival *n* = 88		OR (95% CI)	*p*	SD
Male, *n* (%)	69	(78.4)	69	(78.4)	1.0 (0.49–2.05)	1.000	0.00%
Age, age	53.5	±20.8	52.5	±18.2	-	0.734	5.12%
Comorbidities							
CVA, *n* (%)	0	(0.0)	0	(0.0)	-	-	-
HTN, *n* (%)	9	(10.2)	9	(1.2)	1.0 (0.38–2.65)	1.000	0.00%
CAD, *n* (%)	1	(1.1)	1	(1.1)	1.0 (0.06–16.24)	1.000	0.00%
CHF, *n* (%)	0	(0.0)	0	(0.0)	-	-	-
DM, *n* (%)	9	(10.2)	9	(10.2)	1.0 (0.38–2.65)	1.000	0.00%
ESRD, *n* (%)	0	(0.0)	0	(0.0)	-	-	-
GCS, *n* (%)	6.5	±4.5	7.02	±4.1	-	0.382	−13.22%
ISS	34.6	±7.1	34.4	±7.7	-	0.863	2.60%
Monocytes (10^6^/L)	594	±385	580	±361	-	0.805	-
Neutrophils (10^6^/L)	9316	±6507	9930	±5415	-	0.497	-
Platelets (10^6^/L)	238,284	±78,318	231,761	±75,263	-	0.574	-
Lymphocytes (10^6^/L)	2214	±1372	1807	±1162	-	0.036	-
MLR	0.2	±0.5	0.3	±0.5	-	0.762	-
NLR	6.4	±7.1	7.5	±6.3	-	0.261	-
PLR	145.8	±100.2	157.6	±75.8	-	0.378	-

CAD = coronary artery disease; CHF = congestive heart failure; CI = confidence interval; CVA = cerebral vascular accident; DM = diabetes mellitus; ESRD = end-stage renal disease; GCS = Glasgow Coma Scale; HTN = hypertension; ISS = injury severity score; MLR = monocyte to lymphocyte ratio; NLR = neutrophil to lymphocyte ratio; OR= odds ratio; PLR = platelet-to-lymphocyte ratio; SD = Standardized Difference between matched cohorts.

**Table 3:** Results of the univariate and multivariate analyses to identify independent risk factors for mortality in patients with polytrauma.

Variables	Univariate Analysis			Multivariable Analysis		
	OR	95% CI	*p*	OR	95% CI	*p*
Age	1.0	(0.99–1.01)	0.486	1.0	(1.01–1.05)	<0.001
Male	1.5	(0.96–2.42)	0.075	1.1	(0.62–1.99)	0.721
HTN	1.8	(1.10–3.05)	0.019	0.5	(0.26–0.98)	0.042
GCS	0.8	(0.75–0.84)	<0.001	0.8	(0.76–0.86)	<0.001
ISS	1.1	(1.09–1.16)	<0.001	1.1	(1.07–1.14)	<0.001
Monocytes	1.0	(0.93–1.05)	0.598	1.0	(0.84–1.16)	0.880
Neutrophils	1.0	(0.99–1.00)	0.441	1.0	(0.99–1.01)	0.556
Platelets	1.0	(0.99–1.05)	0.148	1.0	(0.97–1.07)	0.512
Lymphocytes	1.0	(0.99–1.04)	0.099	1.0	(1.00–1.06)	0.043
MLR	0.9	(0.49–1.68)	0.745	0.9	(0.10–7.43)	0.883
NLR	1.0	(0.98–1.03)	0.772	1.0	(0.94–1.11)	0.599
PLR	1.0	(0.98–1.03)	0.728	1.0	(0.95–1.06)	0.948

CI = confidence interval; GCS = Glasgow Coma Scale; HTN = hypertension; ISS = injury severity score; MLR = monocyte to lymphocyte ratio; NLR = neutrophil to lymphocyte ratio; OR= odds ratio; PLR = platelet-to-lymphocyte ratio.
